# Longitudinal follow up of dementia prevalence and risk in First Nations Australians living in the Torres Strait

**DOI:** 10.1002/alz70860_104039

**Published:** 2025-12-23

**Authors:** Sarah G Russell, Rachel Quigley, Edward Strivens, Rhiann Sue See

**Affiliations:** ^1^ James Cook University, Cairns, QLD, Australia; ^2^ James Cook University, Smithfield, QLD, Australia

## Abstract

**Background:**

Previous research by the team identified a threefold prevalence of dementia in First Nations Australia living in the Torres Strait aged 45 and over. A total of 274 people participated in the study with chronic disease being a key dementia risk factor. Based on the Lancet Commission modelling, we used the prevalence study data to model the population attributable risk for dementia in the Torres Strait. Findings suggested almost 40% of risk were associated with modifiable risk factors. In order to better understand these risk factors, a longitudinal follow has been completed with the original cohort ten years later.

**Method:**

Original participants in the prevalence study were invited to have a comprehensive geriatric review. This included a diagnostic medical examination assessing physical, cognitive, and psychosocial factors together with culturally appropriate cognitive screening. Data were also collected on existing and emerging risk factors and associated problems of ageing including hearing, vision, frailty, depression and anxiety, nutrition, pain, sleep, and quality of life. Participants were classified as having normal cognition, Cognitive Impairment No Dementia (CIND) or dementia.

**Result:**

With over 80% of the sample reviewed, results to date show a high mortality rate, with 50% of the sample deceased. Within the remaining sample, 15% had progressed from normal cognitive status to either CIND or dementia. Of those classified as CIND previously, 4% remained stable with CIND and 10% progressed to dementia. 85% of the original sample of those diagnosed with dementia are now deceased. There were no significant age differences between groups (*p*> .05) highlighting the impact of dementia and cognitive impairment within younger groups in First Nations communities. Modifiable risk factors including vascular risk factors were significantly associated with cognitive impairment (*p*> .05).

**Conclusion:**

Understanding the fundamental factors influencing dementia risk in First Nations in Australia is essential to enable the team to develop and test culturally appropriate interventions and preventative strategies that will reduce the burden of chronic disease and geriatric conditions in First Nations Australians.

